# The role of high mobility group box chromosomal protein 1 expression in the differential diagnosis of hepatic actinomycosis: a case report

**DOI:** 10.1186/1752-1947-7-31

**Published:** 2013-01-25

**Authors:** Chuan-Xin Wu, Hui Guo, Jian-Ping Gong, Qi Liu, Hang Sun

**Affiliations:** 1 Department of Hepatobiliary Surgery, The Second Affiliated Hospital of Chongqing Medical University, Chongqing 400010, China; 2 Key Laboratory of Molecular Biology for Infectious Diseases, Ministry of Education, Liver Diseases Research and Treatment Center, The Second Affiliated Hospital of Chongqing Medical University, Chongqing 400010, China

**Keywords:** Diagnosis, Hepatic actinomycosis, High mobility group box chromosomal protein 1, Tumor marker

## Abstract

**Introduction:**

Primary hepatic actinomycosis is a rare disease, but is important in the differential diagnosis of hepatoma in endemic areas. As high mobility group box chromosomal protein 1 plays an important role in the pathogenesis of both acute and chronic inflammatory conditions, we postulate that high mobility group box chromosomal protein 1 may have a possible pathogenic role in hepatic actinomycosis. To the best of our knowledge, our report is the first to detect an association between highly elevated high mobility group box chromosomal protein 1 expression and hepatic actinomycosis.

**Case presentation:**

A 67-year-old Chinese man was admitted to our hospital with a three-month history of epigastric pain, anorexia, and subjective weight loss. Ultrasonography and computed tomography of the patient’s abdomen confirmed a hypodense mass measuring seven cm in diameter in the left lateral segment of his liver. A hepatic tumor was suspected and surgical resection was scheduled. Histopathologic examination revealed that the overall features of the hepatic tissues were consistent with hepatic actinomycosis. Whole blood and hepatic tissue samples of the patient, of patients who had hepatocellular carcinoma and of healthy donors were collected. Serum high mobility group box chromosomal protein 1 concentration in actinomycosis was 8.5ng/mL, which was higher than the hepatocellular carcinoma level of 5.2ng/mL and the normal level of <three ng/mL. High mobility group box chromosomal protein 1 messenger ribonucleic acid levels and high mobility group box chromosomal protein 1 protein content in the affected tissues of this patient with hepatic actinomycosis were higher than those of the control and hepatocellular carcinoma tissues. The results of immunohistochemistry showed the following: in the control tissues, high mobility group box chromosomal protein 1 was distributed mainly in the cytoplasm; in the hepatocellular carcinoma tissues, high mobility group box chromosomal protein 1 was distributed primarily in the nucleus; and in the actinomycosis tissues, high mobility group box chromosomal protein 1 was increased in both the cytoplasm and nucleus.

**Conclusion:**

High mobility group box chromosomal protein 1 may have a potent biological effect on the pathogenesis of hepatic actinomycosis as a novel cytokine and may be a useful marker in the differential diagnosis of hepatic actinomycosis.

## Introduction

Actinomycosis is a chronic, slowly progressive infection caused by the Gram-positive anaerobic bacterium *Actinomyces israelii*. *A. israelii* is part of the normal flora of the oropharynx, gastrointestinal tract, and female genital tract [1]. Although actinomycosis is usually manifested as abscesses of the cervicofacial region, any site or system of the body can be involved, including the thorax, abdomen, pelvis, and central nervous system. Hepatic involvement with actinomycosis has been reported in 15% of patients with abdominal infections, representing 5% of all cases of actinomycosis [2]. Primary hepatic actinomycosis is a very rare condition and should be considered if there are no signs of primary involvement of the abdominal area or any other area of the body. Hepatic actinomycosis can mimic a hepatic tumor [3]. To date, there is no single tumor marker that can be used to differentiate hepatic actinomycosis from liver tumors.

High mobility group box chromosomal protein 1 (HMGB1), a nuclear deoxyribonucleic acid (DNA)-binding protein, was recently rediscovered as a new potent pro-inflammatory cytokine when present extracellularly [4]. A growing number of scientific reports describe the presence of extracellular and cytoplasmic HMGB1 in patients with various inflammatory conditions, including acute as well as chronic ones. Extracellular HMGB1 induces the production of pro-inflammatory cytokines in macrophages. When released by activated monocytes, HMBG1 activates the release of downstream cytokines that lead to cell death. Further, like other cytokine mediators of endotoxemia, HMGB1 activates human monocytes to release pro-inflammatory cytokines [5]. Therefore, it plays an important role in the pathogenesis of both acute and chronic inflammatory conditions. As hepatic actinomycosis is considered a type of chronic inflammatory condition, we postulate that HMGB1 plays a role in the pathogenesis of hepatic actinomycosis. Here we detected the expression of HMGB1 in a patient with hepatic actinomycosis and propose that HMGB1 may be a useful marker in differentiating primary hepatic actinomycosis from malignancy.

## Case presentation

A 67-year-old Chinese man was admitted to our hospital with a three-month history of epigastric pain, anorexia, and subjective weight loss. His previous medical history was unremarkable. The patient did not present any associated fever or chills. He reported that his bowel habits were normal, with no blood or mucus in the stool. The patient reported that he did not smoke or drink. On examination, the patient was afebrile, with no pallor, jaundice, or lymphadenopathy. An abdominal examination revealed that he had an enlarged liver. A whole blood sample was collected. Laboratory test results were as follows: hemoglobin, 128g/L (normal, 110 to 160g/L); white blood cell count, 7.5×10^9^/L (normal, four to 10.0×10^9^/L); platelet count, 125×10^9^/L (normal, 99 to 303×10^9^/L); serum bilirubin, 18μmol/L (normal, 2.3 to 20.4μmol/L); aspartate aminotransferase, 18U/L (normal, five to 34U/L); and alanine aminotransferase, nine U/L (normal, 0 to 40U/L). Blood serologies for hepatitis B and C and human immunodeficiency virus were negative. The serum alpha-fetoprotein concentration was 17.4ng/mL (normal, <20ng/mL), the serum carcinoembryonic antigen concentration was 2.8ng/mL (normal, <five ng/mL).

**Figure 1:**
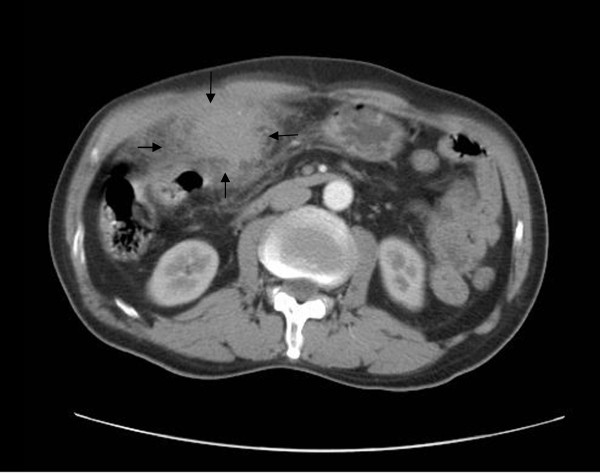
**Abdominal computed tomography.** Contrast-enhanced computed tomography during the arterial phase shows a hypodense lesion (arrows) in the left lobe of the liver, with infiltration to the surrounding tissue.

Ultrasonography of the patient’s abdomen showed a mass in his left liver lobe that measured approximately seven cm in diameter. Computed tomography of his abdomen confirmed a mass measuring seven cm in diameter in the left lateral segment of his liver (Figure 1). The tumor was relatively poorly enhanced. The surface of the left liver lobe was blurred, leading to suspicion of tumor infiltration to the surrounding tissue. No other abnormality was detected in the patient’s abdomen. The upper and lower gastrointestinal series and chest radiography results were normal. A hepatic tumor was suspected and surgical resection was scheduled. Intra-operatively, a firm mass (seven cm in diameter) at the base of segments II and III was noted. The suspected tumor was adhered to the greater omentum, transverse colon, stomach, and anterior abdominal wall. A left lateral hepatectomy and distal gastrectomy were performed. Hepatic tissue three cm away from the lesion was considered a control and hepatocellular carcinoma (HCC) tissues were obtained from the patients with HCC who accepted surgical resection. Tissue specimens from the liver were collected and stored in liquid nitrogen until they were examined histopathologically.

**Figure 2:**
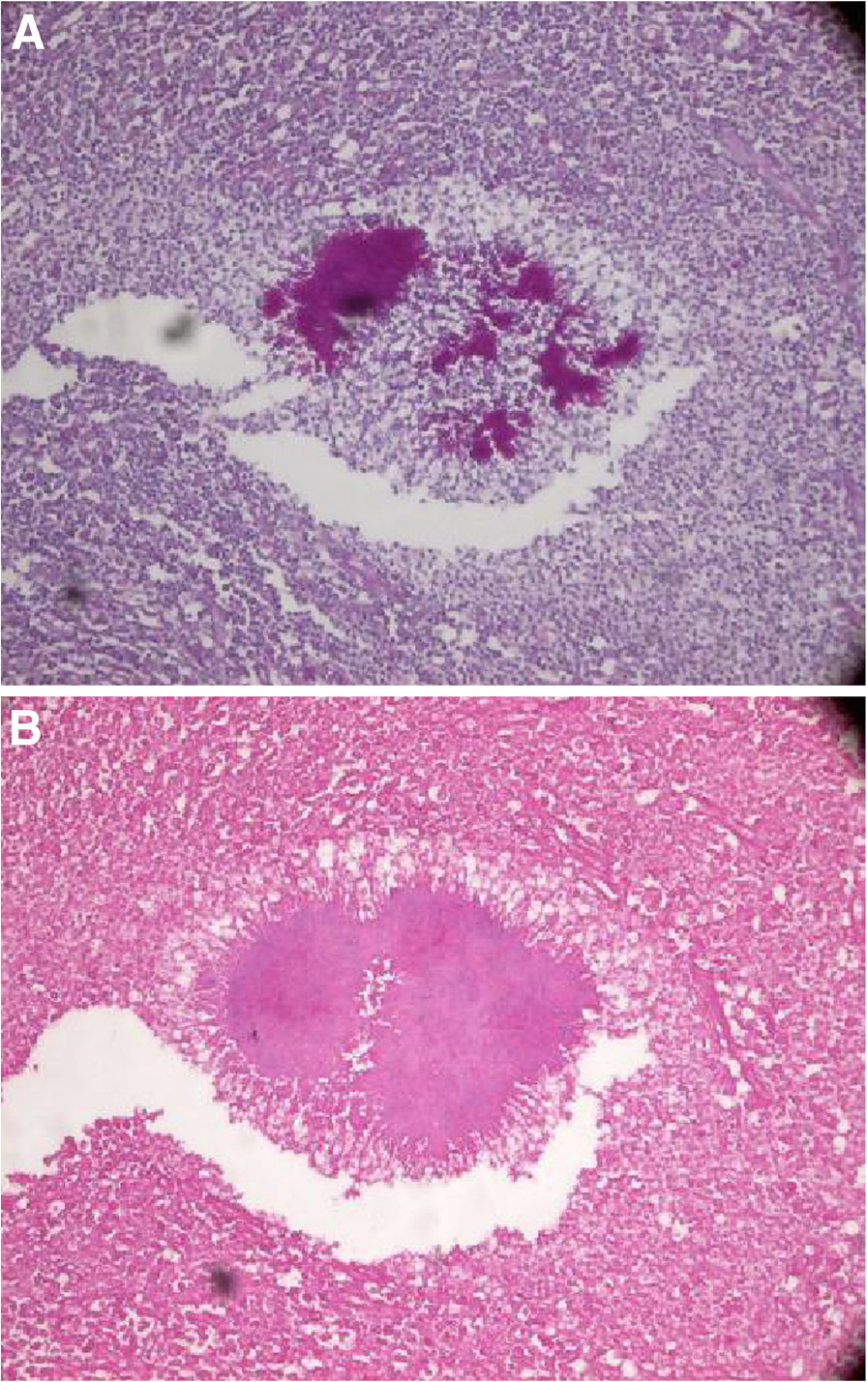
**Pathological section demonstrating the characteristic sulfur granules.** (**A**) Liver tissue showing sulfur granules surrounded by neutrophils, foaming histiocytes, lymphocytes, and plasma cells (hematoxylin-eosin, ×200). (**B**) Bacterial colonies of *Actinomyces* tested positive for periodic acid-Schiff with the Splendore–Hoeppli reaction at the periphery and outer zone of the inflammatory cells (×200).

On pathological examination, the resected liver appeared as a yellow-white mass that contained a number of pus-like areas; several smaller nodules were adjacent to the mass. The histopathologic examination revealed prominent lobular inflammation, with large areas of hepatocyte loss and necrosis. Bacterial colonies of *Actinomyces* were observed surrounded by polymorphs as revealed by hematoxylin-eosin staining. The colonies tested positive for periodic acid-Schiff with the Splendore–Hoeppli reaction at the periphery and outer zone of inflammatory cells (Figure 2). No evidence of malignancy was found. The overall features were those of hepatic actinomycosis. The abscess involved the stomach and extended to the gastric muscularis propria, leading to focal fibrosis and inflammation.

**Figure 3:**
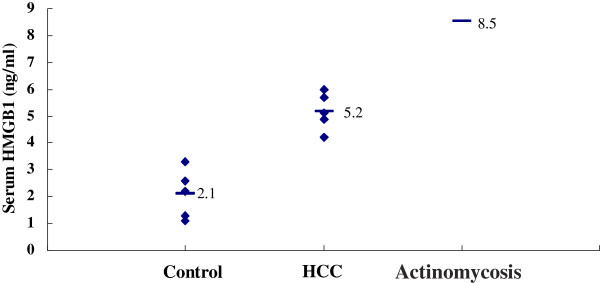
**Serum levels of high mobility group box chromosomal protein 1.** Serum levels of high mobility group box chromosomal protein 1 were evaluated by enzyme-linked immunosorbent assay. Bars show the means. The high mobility group box chromosomal protein 1 level in the patient with actinomycosis was higher than the control and hepatocellular carcinoma groups, and the serum high mobility group box chromosomal protein 1 levels in the hepatocellular carcinoma group were significantly higher than the control group (5.2±0.7ng/mL vs. 2.1±0.9ng/mL; P<0.01). HMGB1, high mobility group box chromosomal protein 1; HCC, hepatocellular carcinoma.

The serum HMGB1 levels were measured by enzyme-linked immunosorbent assay. The serum HMGB1 concentration in the patient who had actinomycosis was 8.5ng/mL [6,7], 2.1±0.9ng/mL in the control group and 5.2±0.7ng/mL in the HCC group. The HMGB1 level in the patient who had actinomycosis was higher than the control and HCC groups (Figure 3).

**Figure 4:**
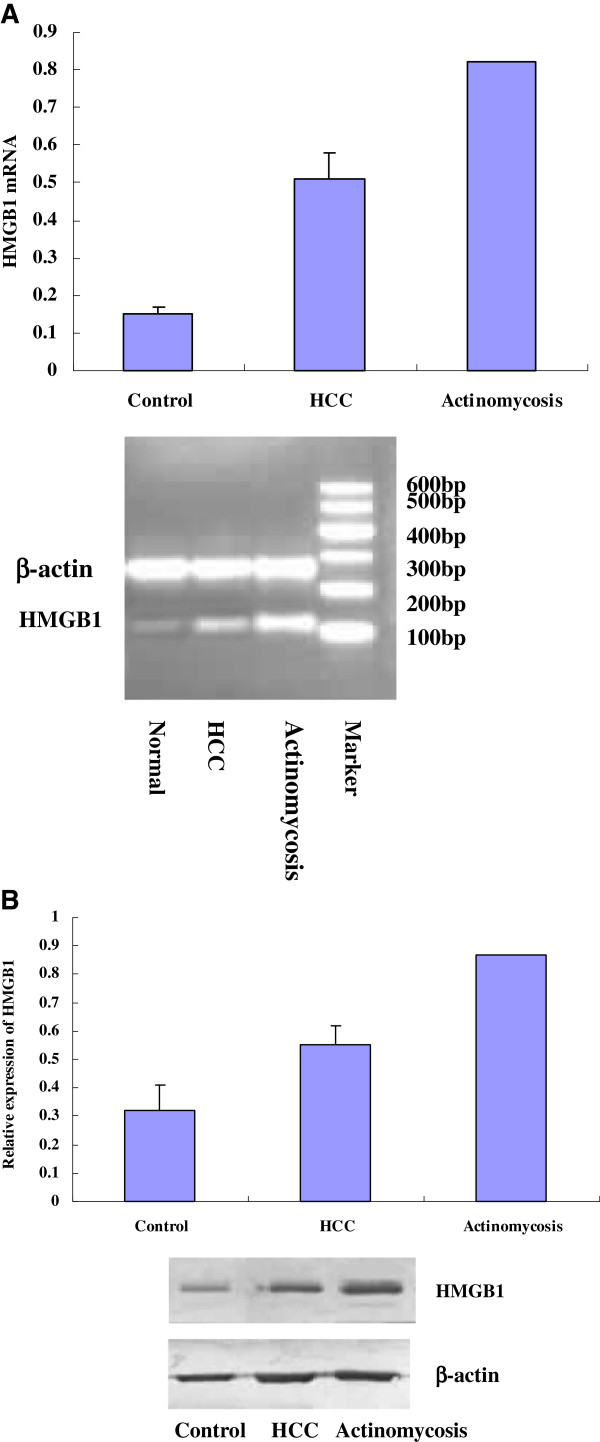
**Expression of high mobility group box chromosomal protein 1 in the hepatic tissues.** (**A**) High mobility group box chromosomal protein 1 messenger ribonucleic acid levels were determined by reverse transcriptase polymerase chain reaction. The high mobility group box chromosomal protein 1 messenger ribonucleic acid levels in the affected tissues of the patient with hepatic actinomycosis were significantly higher than those in the control and hepatocellular carcinoma tissues (0.82 vs. 0.15±0.02 and 0.51±0.07, respectively). (**B**) Total protein was extracted from hepatic tissues and high mobility group box chromosomal protein 1 content was detected by western blot. The high mobility group box chromosomal protein 1 content in the affected tissues of the patient with hepatic actinomycosis was significantly higher than that of the control and hepatocellular carcinoma tissues (0.87 versus 0.32±0.09 and 0.55±0.07, respectively). HMGB1, high mobility group box chromosomal protein 1; HCC, hepatocellular carcinoma; mRNA, messenger ribonucleic acid; bp, base pairs.

Expression of HMGB1 messenger ribonucleic acid (mRNA) and protein in hepatic tissues was determined by reverse transcriptase polymerase chain reaction and western blot, respectively. HMGB1 mRNA and protein levels were significantly increased in the hepatic actinomycosis tissue compared with the control and HCC tissue (Figure 4).

**Figure 5:**
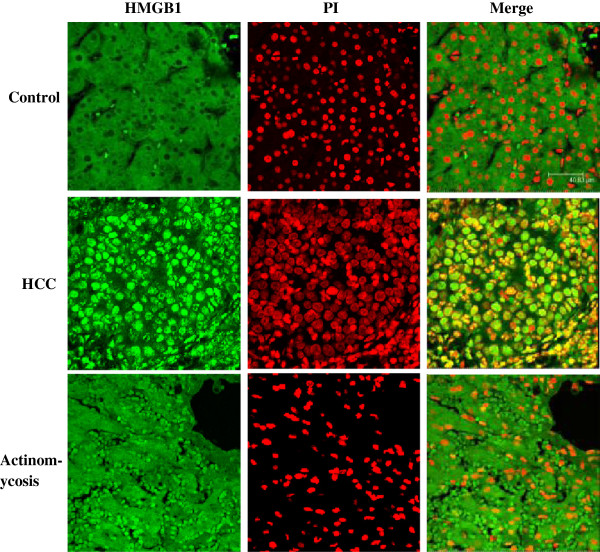
**Intracellular localization of high mobility group box chromosomal protein 1 in the hepatic tissues.** Cells were double-stained with anti-high mobility group box chromosomal protein 1 antibodies and propidium iodide and analyzed by confocal microscopy. In the control cells, high mobility group box chromosomal protein 1 was located in the cytoplasm. In the hepatocellular carcinoma group, high mobility group box chromosomal protein 1 was mainly located in the nucleus. In the actinomycosis-affected cells, a high level of high mobility group box chromosomal protein 1 was found in both the cytoplasm and nucleus (original magnification, ×400). HMGB1, high mobility group box chromosomal protein 1; HCC, hepatocellular carcinoma; PI, propidium iodide.

Immunocytochemistry and confocal laser-scanning microscopy were used to confirm the distribution of HMGB1 protein in the hepatic tissues. Cells were double-stained with anti-HMGB1 antibodies and propidium iodide and analyzed by confocal microscopy. The cytoplasm of the control cells showed intense green staining, whereas the nucleus showed weak staining, indicating that HMGB1 is distributed mainly in the cytoplasm. In the HCC group, the nucleus stained very strongly, indicating that HMGB1 is distributed mainly in the nucleus. In the actinomycosis-affected cells, staining in both the cytoplasm and nucleus was enhanced, suggesting increased HMGB1 protein levels throughout the hepatic cell in this patient with primary hepatic actinomycosis (Figure 5).

The patient was treated with penicillin (intravenous penicillin G, 24 million units per day for the first two weeks and oral penicillin V, 1.5g per day for the following six months) and discharged when he was asymptomatic. He remained healthy, and the serum HMGB1 concentration was 0.92ng/mL (normal, <three ng/mL) at two years after surgery.

## Discussion

Primary hepatic actinomycosis is a rare disease that should be considered in the differential diagnosis of liver masses. The symptoms are usually non-specific and include upper abdominal pain, weight loss, a palpable mass, and low-grade fever (or no fever). Microcystic anemia and hypoalbuminemia are common features of hepatic actinomycosis. However, such symptoms are also observed in malignant disorders [8]. As imaging often leads to a suspicion of neoplasms and positive cultures are notoriously difficult to obtain, the pre-operative rate of diagnosis of hepatic actinomycosis is <10%. Therefore, an exploratory laparotomy is usually required for a definitive diagnosis of hepatic actinomycosis [9]. Definitive diagnosis is based on histochemical, macroscopic, and microscopic examination of surgical tissue specimens, which reveal yellow sulfur granules and basophilic filament aggregates [9]. Our patient presented with abdominal pain and prominent weight loss, but the blood test results were all within normal limits. A radiologic examination showed a solid liver mass mimicking a hepatic neoplasm. A hepatic tumor was suspected and surgical resection was performed. Histopathologic examination revealed an abscess containing multiple collections of heavy acute and chronic inflammatory infiltrates with florid fibrosis. A few clusters of filamentous microorganisms consistent with *Actinomyces* colonies were present, and the diagnosis of hepatic actinomycosis was confirmed.

There is no single tumor marker that can be used to differentiate hepatic actinomycosis from liver tumors. Several studies have shown an association between hepatic actinomycosis and elevated CA19-9 levels. CA19-9 is a carbohydrate epitope that is sialylated on the surface of various tumors. CA19-9 is not organ specific and is clinically used as a marker for pancreatic, hepatobiliary, and gastric malignancies. The CA19-9 level is often elevated in benign diseases of the hepatobiliary system, renal failure, pleural effusion, intestinal pneumonia, and systemic lupus erythematosus. However, the level of elevation in these benign conditions is often lower than that in malignant conditions [9,10]. Despite these benefits, CA19-9 has inadequate sensitivity and specificity to be used as a marker for differentiating hepatic actinomycosis from liver tumors [11,12].

HMGB1 is located in the nucleus of most cells. It has been reported that HMGB1 is translocated from the nucleus to the cytosol and then released extracellularly. An increasing number of scientific reports have described the presence of extracellular and cytoplasmic HMGB1 in various inflammatory conditions. With respect to acute inflammation, HMGB1 has been demonstrated to be of pathogenic relevance in sepsis, pneumonia, and endotoxemia. Serum levels of HMGB1 significantly increase 16 to 32 hours following systemic lipopolysaccharide challenge in mice, and a systemic injection of the recombinant HMGB1 in mice is lethal, two findings that provide further support for the pathogenic role of HMGB1 in endotoxemia [4]. Induction of experimental sepsis in mice by cecal ligation and puncture also results in increased serum levels of HMGB1. Sera collected from patients with sepsis demonstrated increased levels of HMGB1 compared with sera from healthy controls, with the highest increase of HMGB1 levels in patients who succumbed to the disease [4]. Intra-tracheal administration of HMGB1 in mice induces acute lung inflammation, with accumulation of neutrophils, edema, and production of pro-inflammatory cytokines [13]. An increase in serum HMGB1 levels has also been reported in patients with hemorrhagic shock [14]. HMGB1 has also been reported to play a possible pathogenic role in chronic inflammatory conditions. The presence of cytoplasmic and extracellular HMGB1 has been reported in experimental arthritis models as well as in rheumatoid arthritis (RA) in humans. HMGB1 has been detected in the majority of investigated synovial fluid samples from patients with RA [5]. In muscle biopsies from patients with chronic myositis, HMGB1 is detected cytoplasmically in muscle fibers, in inflammatory infiltrates, and in small vessel endothelial cells. Biopsies obtained before and after systemic corticosteroid treatment revealed diminished extranuclear HMBG1 expression [15].

In the current study, we showed that serum HMGB1 levels were increased in patients with HCC in comparison to healthy controls, which is consistent with the report of Cheng *et al*. [16]. However, the serum HMGB1 level in the patient who had actinomycosis was significantly higher than the level in the patients who had HCC. We also detected HMGB1 mRNA and protein levels in the hepatic tissues. The results showed that the HMGB1 protein content and mRNA levels were higher in the affected tissue than HCC and the control tissues. Although the expression of HMGB1 increased in the patients who had HCC and the patient with actinomycosis, the distribution of HMGB1 was different. Based on immunohistochemistry, we clearly showed that HMGB1 was distributed mainly in the nucleus in the patients who had HCC, but in the patient with actinomycosis the expression of HMGB1 increased in the cytoplasm and nucleus. These differences helped to identify hepatic tumors and hepatic actinomycosis; however, we need more samples of hepatic actinomycosis to verify our findings.

To the best of our knowledge, our report is the first to detect an association between highly elevated HMGB1 expression and hepatic actinomycosis. As a new pro-inflammatory cytokine, HMGB1 may play a pathogenic role in hepatic actinomycosis and might be a useful marker in the differential diagnosis of hepatic actinomycosis, particularly in patients for whom diagnosis is difficult.

## Conclusions

In summary, primary hepatic actinomycosis is a rare disease, but is important in the differential diagnosis of hepatoma in endemic areas. The expression of HMGB1 in hepatic actinomycosis was significantly high. HMGB1 may be useful in the differential diagnosis of hepatic actinomycosis.

## Consent

Written informed consent was obtained from the patient for publication of this manuscript and accompanying images. A copy of the written consent is available for review by the Editor-in-Chief of this journal. The study protocol was approved by the local Institutional Ethics Committee.

## Competing interests

The authors declare that they have no competing interests.

## Authors’ contributions

CXW and HS performed the research. JPG and QL designed the study. HG performed the pathological analysis, and HS wrote the manuscript. All authors read and approved the final manuscript.

## References

[B1] Wong JJ Kinney TB Miller FJ Rivera-Sanfeliz G Hepatic actinomycotic abscesses: diagnosis and management AJR Am J Roentgenol 2006 186 174 176 10.2214/AJR.04.1691 16357398

[B2] Kocabay G Cagatay A Eraksoy H Tiryaki B Alper A Calangu S A case of isolated hepatic actinomycosis causing right pulmonary empyema Chin Med J (Engl) 2006 119 1133 1135 16834936

[B3] Tamsel S Demirpolat G Killi R Elmas N [Primary hepatic actinomycosis: a case of inflammatory pseudotumor (case report)] Tani Girisim Radyol 2004 10 154 157 15236133

[B4] Wang H Bloom O Zhang M Vishnubhakat JM Ombrellino M Che J Frazier A Yang H Ivanova S Borovikova L Manogue KR Faist E Abraham E Andersson J Andersson U Molina PE Abumrad NN Sama A Tracey KJ HMG-1 as a late mediator of endotoxin lethality in mice Science 1999 285 248 251 10.1126/science.285.5425.248 10398600

[B5] Pullerits R Urbonaviciute V Voll RE Forsblad-D’Elia H Carlsten H Serum levels of HMGB1 in postmenopausal patients with rheumatoid arthritis: associations with proinflammatory cytokines, acute-phase reactants, and clinical disease characteristics J Rheumatol 2011 38 1523 1525 21724730 10.3899/jrheum.110091

[B6] Henes FO Chen Y Bley TA Fabel M Both M Herrmann K Csernok E Gross WL Moosig F Correlation of serum level of high mobility group box 1 with the burden of granulomatous inflammation in granulomatosis with polyangiitis (Wegener’s) Ann Rheum Dis 2011 70 1926 1929 10.1136/ard.2010.146456 21765168

[B7] Abdulahad DA Westra J Bijzet J Limburg PC Kallenberg CG Bijl M High mobility group box 1 (HMGB1) and anti-HMGB1 antibodies and their relation to disease characteristics in systemic lupus erythematosus Arthritis Res Ther 2011 13 R71 10.1186/ar3332 21548924 PMC3218880

[B8] Yu CY Chang WC Gao HW Chao TY Huang GS Hsieh CB Metastatic hepatic actinomycosis Am J Med 2010 123 e9 e11 20800141 10.1016/j.amjmed.2010.01.032

[B9] Lall T Shehab TM Valenstein P Isolated hepatic actinomycosis: a case report J Med Case Reports 2010 4 45 10.1186/1752-1947-4-45 PMC2833155 20181118

[B10] Soardo G Basan L Intini S Avellini C Sechi LA Elevated serum CA 19-9 in hepatic actinomycosis Scand J Gastroenterol 2005 40 1372 1373 10.1080/00365520510024232 16334448

[B11] Buxbaum JL Eloubeidi MA Molecular and clinical markers of pancreas cancer JOP 2010 11 536 544 21068484

[B12] Peng NF Li LQ Qin X Guo Y Peng T Xiao KY Chen XG Yang YF Su ZX Chen B Su M Qi LN Evaluation of risk factors and clinicopathologic features for intrahepatic cholangiocarcinoma in Southern China: a possible role of hepatitis B virus Ann Surg Oncol 2011 18 1258 1266 10.1245/s10434-010-1458-5 21207172

[B13] Lutz W Stetkiewicz J High mobility group box 1 protein as a late-acting mediator of acute lung inflammation Int J Occup Med Environ Health 2004 17 245 254 15387080

[B14] Goldstein RS Gallowitsch-Puerta M Yang L Rosas-Ballina M Huston JM Czura CJ Lee DC Ward MF Bruchfeld AN Wang H Lesser ML Church AL Litroff AH Sama AE Tracey KJ Elevated high-mobility group box 1 levels in patients with cerebral and myocardial ischemia Shock 2006 25 571 574 10.1097/01.shk.0000209540.99176.72 16721263

[B15] Ulfgren AK Grundtman C Borg K Alexanderson H Andersson U Harris HE Lundberg IE Down-regulation of the aberrant expression of the inflammation mediator high mobility group box chromosomal protein 1 in muscle tissue of patients with polymyositis and dermatomyositis treated with corticosteroids Arthritis Rheum 2004 50 1586 1594 10.1002/art.20220 15146429

[B16] Cheng BQ Jia CQ Liu CT Lu XF Zhong N Zhang ZL Fan W Li YQ Serum high mobility group box chromosomal protein 1 is associated with clinicopathologic features in patients with hepatocellular carcinoma Dig Liver Dis 2008 40 446 452 10.1016/j.dld.2007.11.024 18294942

